# Covalent polyphenols-proteins interactions in food processing: formation mechanisms, quantification methods, bioactive effects, and applications

**DOI:** 10.3389/fnut.2024.1371401

**Published:** 2024-03-06

**Authors:** Kangyi Zhang, Jinbao Huang, Dongxu Wang, Xiaochun Wan, Yijun Wang

**Affiliations:** ^1^State Key Laboratory of Tea Plant Biology and Utilization, Key Laboratory of Food Nutrition and Safety, School of Tea and Food Science and Technology, Anhui Agricultural University, Hefei, China; ^2^Joint Research Center for Food Nutrition and Health of IHM, Anhui Agricultural University, Hefei, China; ^3^New-style Industrial Tea Beverage Green Manufacturing Joint Laboratory of Anhui Province, Anhui Agricultural University, Hefei, China; ^4^School of Grain Science and Technology, Jiangsu University of Science and Technology, Zhenjiang, China

**Keywords:** food functional ingredients, covalent interaction, protein, polyphenols, functional foods

## Abstract

Proteins and polyphenols are abundant in the daily diet of humans and their interactions influence, among other things, the texture, flavor, and bioaccessibility of food. There are two types of interactions between them: non-covalent interactions and covalent interactions, the latter being irreversible and more powerful. In this review, we systematically summarized advances in the investigation of possible mechanism underlying covalent polyphenols-proteins interaction in food processing, effect of different processing methods on covalent interaction, methods for characterizing covalent complexes, and impacts of covalent interactions on protein structure, function and nutritional value, as well as potential bioavailability of polyphenols. In terms of health promotion of the prepared covalent complexes, health effects such as antioxidant, hypoglycemic, regulation of intestinal microbiota and regulation of allergic reactions have been summarized. Also, the possible applications in food industry, especially as foaming agents, emulsifiers and nanomaterials have also been discussed. In order to offer directions for novel research on their interactions in food systems, nutritional value, and health properties *in vivo*, we considered the present challenges and future perspectives of the topic.

## 1 Introduction

**Figure 1:**
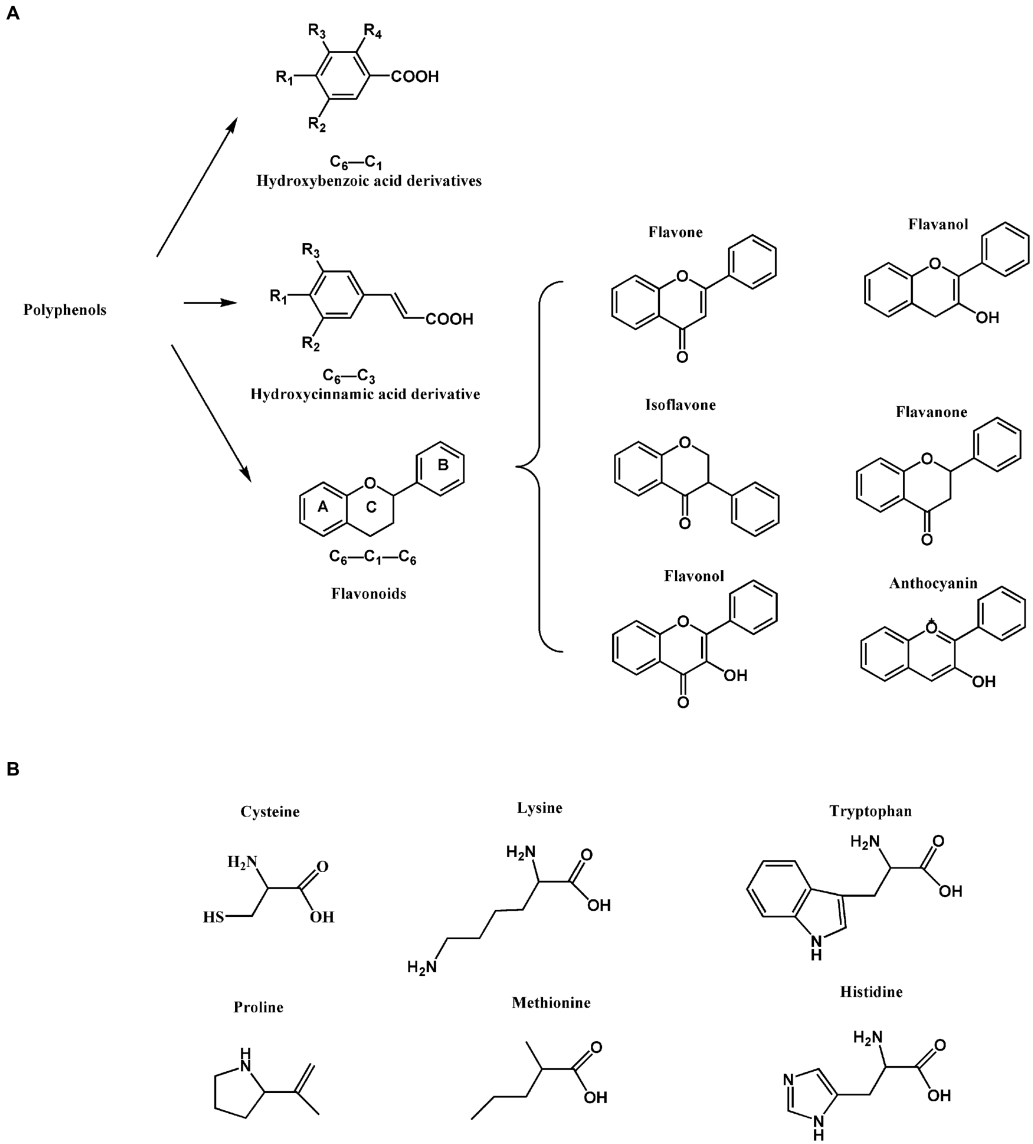
Chemical structures. **(A)** Basic structure of dietary polyphenols. **(B)** Structure of common protein amino acid residues.

In human diet, polyphenols are considered as antioxidants that are mostly abundant and widely distributed active components of plants with high biological activity (1, 2). In this regard, they are known as the “seventh nutrient.” More than 8,000 polyphenolic compounds have been identified, with high levels existing in wine, tea, nuts, berries, cocoa and various plant foods (3, 4). Polyphenols have shown lots of valuable effects and uses in the food industry. First of all, important natural pigments in food industry have been identified to be polyphenols, namely blueberries, cherries, strawberries, anthocyanins, etc. (5, 6). Polyphenols are a general term for plant components that have several phenolic hydroxyl (OH) groups in their molecular structure. Polyphenol compounds contain at least one aromatic ring with OH group, which can be divided into phenolic acid, flavone, flavanol, lignans, etc., in terms of their carbon skeleton structure (7). As shown in Figure 1A, the chemical structures of polyphenolic compounds have three main carbon atom skeletons of C6-C3-C6 (flavonoids), C6-C3 (hydroxycinnamic acid derivatives) and C6-C1 (hydroxybenzoic acid derivatives). *Inter alia*, the most diverse and broadly distributed polyphenols are flavonoid, wherein they include flavonol, flavone, isoflavone, flavanone, anthocyanin and flavanol. With regards to health benefits, polyphenols have demonstrated strong anti-inflammatory, antibacterial and antioxidant properties, and in addition, have been found to possess anti-cancer and anti-cardiovascular disease effects (8–10). Therefore, there is considerable interest in polyphenols as bioactive components in functional foods.

Protein is a biological macromolecule with a certain spatial structure, wherein it is considered as a substance formed by coiled folding of polypeptide chains. Protein is one of the six nutrients required by the human body and is mainly found in livestock and poultry meat, milk, eggs, cereals, legumes and other foods (11, 12). Coexistence of dietary proteins-polyphenols is very common, namely soy milk (soy protein-flavonoids), fruit milk (dairy protein-fruit polyphenols), milk tea (dairy protein-tea polyphenols), and plant protein drinks (plant protein-plant polyphenols). The interaction between polyphenols and proteins influences the structure, taste and function of the food (13–15). Polyphenols form covalent bonds with proteins, mostly with free amino (lysine), sulfhydryl (SH, cysteine), and carbonyl groups as well as other amino acid residues (like tryptophan, proline, methionine, histidine and tyrosine, etc.) in the amino acid side chain of proteins (Figure 1B displays the structures of common amino acid residues).

Proteins and polyphenols in food are highly susceptible to interaction during food storage, transportation and processing, which adversely affect their structure, function, and nutritional value (16–18). Mechanistically, complexes of polyphenols and proteins are formed through interactions that are covalent and noncovalent. Comparatively, covalent bonding is irreversible and stronger than noncovalent bonds, wherein both bonds have a strong influence on the structure and function of proteins and polyphenols. Hence, the complexes formed by covalent bonds are more suitable for food applications. In addition, covalent effects tend to be more effective than non-covalent effects in enhancing the antioxidant activity of polyphenols (19).

During processing, transportation and storage of food, protein-polyphenol interactions should be better understood in order to control the changes in functionality and quality of protein-polyphenol complexes. Hence, the present review presents a systematic overview of the formation mechanism of protein and polyphenol covalent complexes in food and their influencing factors, characterization methods and the functional properties and potential applications, provide a theoretical framework for the high-value utilization of proteins and polyphenols in foods, as well as for product development and application within the food industry.

## 2 Covalent polyphenols-proteins interactions during food processing

Covalent polyphenol-protein interaction is a specific type of binding that occurs under specific conditions. The initiation of covalent bonds between polyphenols and proteins is mainly due to the formation of quinone or semi-quinone radicals (20, 21). During manufacturing of food products, various processing methods are usually used to treat the food products, for instance heat treatment, enzymatic treatment, ultrasound, etc. The processing methods can have a covalent effect on the components of polyphenol-protein in the food system, while their structure, sensory quality and functional properties can be affected. Currently, researchers have partially investigated the effect of processing methods on the covalent interaction between polyphenols and proteins in foods.

### 2.1 Heat treatments

**Figure 2:**
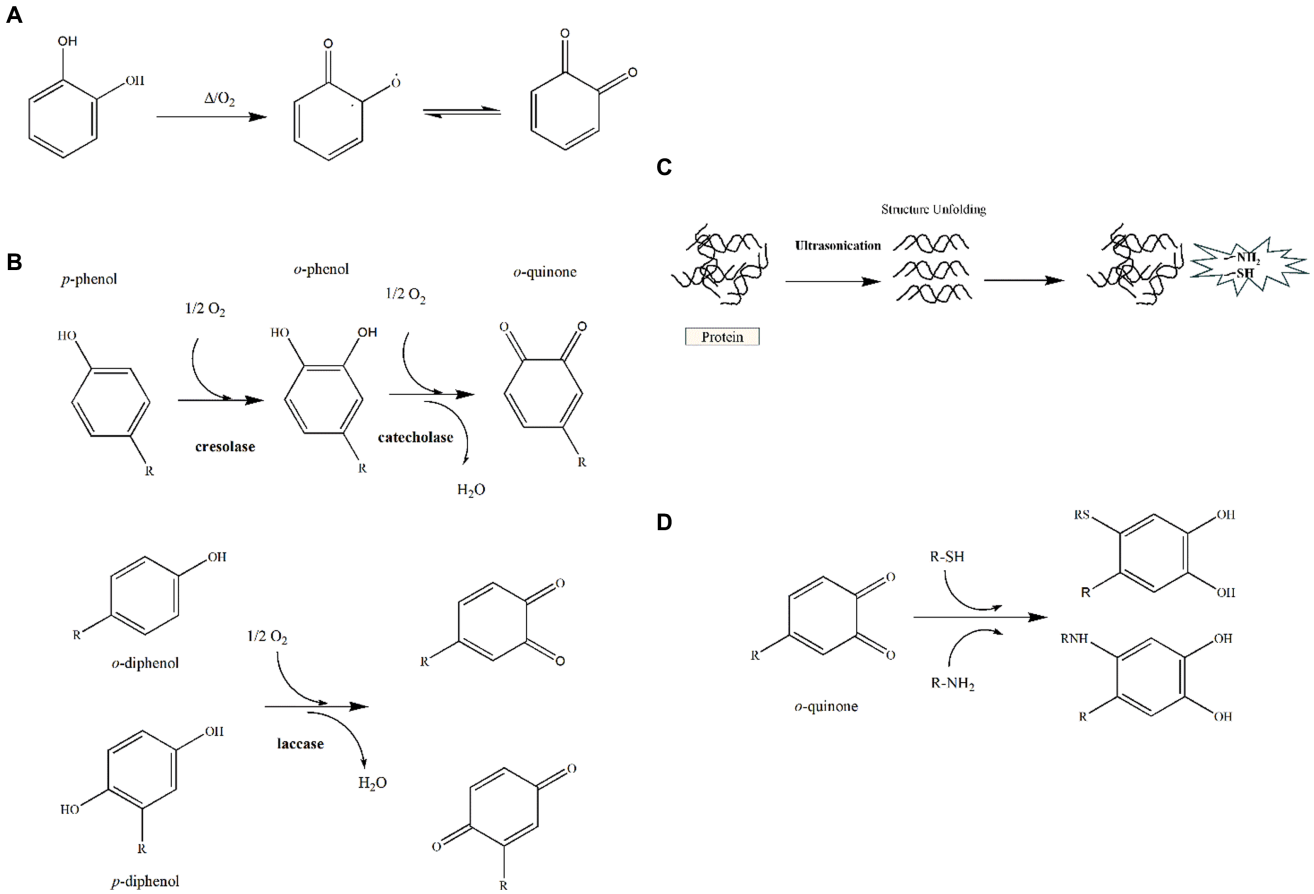
Covalent interactions between polyphenols and proteins. **(A)** Formation of quinone compounds during heat treatment. **(B)** Enzymatic mechanism for formation of o-quinones. **(C)** Schematic representation of protein ultrasound-exposed active groups. **(D)** Reaction of *o*-quinones with thiol and amine groups by nucleophilic 1,4-Michael addition.

During the production process, food products are subjected to different heat treatment processes to improve functional properties, nutritional and organoleptic characteristics. Most importantly, thermal treatment is a crucial process utilized in the food industry for sterilization. Heat treatment normally reduces pathogenic bacteria in food and facilitates long-term storage. Common heat treatments include pasteurization, boiling, baking, autoclaving, and ultra-high temperature (UHT) instantaneous sterilization. Among the common processing methods, heat treatment has been found to induce polyphenols to autoxidize to form quinone or semiquinone structures, which structurally unfolds proteins, thereby exposing more amino acid residues and thus potentially forming irreversible covalent bonds between the two (Figures 2A,D). Kaur et al. (22) found that milk proteins formed higher amounts of covalent binding with phenolic acids in oats after UHT treatment compared to alkali treatment. Chen et al. preheated soybean isolate and found that it exhibited a stronger binding ability to anthocyanin after treatment at 121°C, wherein it could effectively improve the thermal and oxidative stability of anthocyanin (23). Also, wheat alcohol-soluble protein-proanthocyanidin (PA) interactions could help to systematically control foaming and gelling, create new textures, and reduce inflammatory responses. Whereas both are more potent at high temperatures, wheat alcohol-soluble protein-procyanidin interactions can modify protein structure or available binding sites to reduce inflammatory responses to proteins such as allergic or celiac reactions (24).

### 2.2 Enzymatic processing

The oxidation of phenolic compounds in the presence of oxygen is enzymatically catalyzed to form quinone structures. It is well known that enzymatic oxidation requires the simultaneous fulfillment of three conditions, that is oxygen, phenolic compounds and polyphenol oxidase (PPO) (Figure 2B). Due to their electrophilic nature, quinones have the ability to undergo reactions with the free amino groups of proteins, specifically lysine, cysteine, and tryptophan, resulting in the formation of covalent bonds (25–27). Besides, polyphenols can be covalently bound to amino acids containing SH groups upon oxidation. Li et al. prepared covalently coupled compounds of lactoferrin with epigallocatechin gallate (EGCG) using enzymatic and non-enzymatic methods. Enzymatic cross-linking promoted the covalent attachment of EGCG to lactoferrin more effectively and reduced the allergenicity of the protein (28). In addition, in food processing, proteins are often enzymatically modified to enhance their functional properties. However, the functional properties of proteins are altered due to the production of small peptides of low molecular weight, loss or alteration of natural structure, and enhancement of peptide interactions with the peptide itself or with other substances in the matrix during enzymatic digestion. Usually, the products obtained from proteolysis are highly digestible, hypoallergenic and have antioxidant activity (29, 30). Using different soy protein hydrolysates that have been covalently bound to EGCG, it was found that different amino acid compositions and molecular weights affected the soy protein hydrolysate-EGCG interactions and emulsification properties. Meanwhile, peptide from EGCG and soy protein hydrolysates demonstrated a synergistic effect on the emulsification and antioxidant capacity of covalently coupled compounds (31).

### 2.3 Ultrasonication

As a non-thermal treatment technique, ultrasound is widely used in the food industry because of its maneuverability, time-saving and low sensitivity. The chemical and physical properties of materials are improved by the cavitation, micro-fluidization and mechanical effects that are produced by ultrasonication (32). Firstly, the solubility of insoluble proteins can be increased. In addition, the cavitation effect breaks up the droplets and generates lots of OH radicals in solution, while simultaneously, there is mechanical unfolding of protein structure coupled with reaction of exposed SH and amino groups with OH radicals to produce activated protein derivatives, amid covalent interaction with polyphenolic compounds (Figures 2C,D). It was shown that ovalbumin-EGCG (OVA-EGCG) conjugates produced via ultrasound-assisted free radical treatment could be used as potential emulsifiers and antioxidants, thus expanding the application of OVA as a dual functional component (33). This phenomenon could potentially be attributed to the increased molecular flexibility of OVA resulting from the treatment, which in turn, might contribute to the improved emulsification properties. Therefore, more in-depth studies on the effect of processing methods on polyphenol-protein interactions in food components are needed. Chen et al. found that ultrasound treatment helped to accelerate the covalent reaction between myofibrillar proteins and polyphenols, which promoted the unfolding of myofibrillar protein structures, thus resulting in products with better antioxidant activity and digestive properties (34).

### 2.4 pH

Proteins are susceptible to denaturation during food processing, particularly in response to fluctuations in pH levels. This can result in alterations to the protein structure, while polyphenols may generate free radicals or quinones in alkaline environments, leading to their covalent binding with proteins (35, 36). Liu et al. (37) observed variations in the binding affinity of EGCG for β-LG under different pH conditions, with the binding affinities ranking in descending order as pH 7.0 > pH 5.3 > pH 2.5. This discrepancy can be attributed to the structural changes of β-LG, in which the fact that β-LG exists as a dimer at pH 7.0, a tetramer at pH 5.3, and a monomer at pH 2.5. Furthermore, the binding capacity is significantly influenced by the type of polyphenol and the pH conditions. For example, bovine serum proteins bind more to tannins at pH 4.9 than at pH 7.8 (20), while gallic acid is lowest with bovine serum proteins at pH 3.5 (38). Therefore, alterations in pH levels play a crucial role in modulating the interaction between proteins and polyphenols, highlighting the importance of considering pH in the context of the food industry.

Notably, polyphenol-protein covalent interaction reactions are intricate and have the potential to result in the generation of undesirable products. The safety of the product must also undergo evaluation. For example, in a study of sugarcane leaf polyphenol-zearalolysin prepared by a covalent method and evaluated for safety, it was found that the hemolysis rate was less than 5% and the cell viability (LX-2) was greater than 80%, indicating a favorable safety profile (39). Hence, it is imperative for researchers to carefully select the binding mode of polyphenol-protein complexes based on their research objectives and practical application to prevent the generation of undesirable products. In addition, the assessment of potential risks associated with any newly formed covalent should be conducted before widespread use in the food industry.

## 3 Characterization of polyphenol-protein covalent complexes

Different analytical methods are required for characterization of covalent complexes properties like conformation, composition, relative molecular mass, bond type and structure (Table 1) (20, 56). These analytical methods mainly include circular dichroism (CD), differential scanning calorimetry (DSC), electrophoresis, fluorescence spectroscopy, Fourier transform infrared spectroscopy (FT-IR), reverse phase high performance liquid chromatography, light scattering, mass spectrometry (MS), nuclear magnetic resonance (NMR), UV–Vis absorption spectroscopy, volume exclusion chromatography and small-angle X-ray scattering (20, 57, 58). The nature of polyphenols that form complexes with proteins via these analytical methods can be better understood.

**Table 1:** Characterization of polyphenol-protein covalent complexes.

	Method of analysis	Characteristics	References
Electrophoresis	CE	Differentiate free proteins from protein-polyphenol covalent complexes	(40, 41)
	SDS-PAGE		
Mass spectrometry	MALDI-MS	Molecular weight and structure	(42–44)
	ESI-MS		
Spectroscopy	FT-IR	Changes in protein structure	(45–47)
	CD		
	Fluorescence spectroscopy		
Chromatography	SEC	Molecular mass	(48, 49)
	RP-HPLC		
Light scattering	SLS	Physicochemical properties	(50, 51)
	DLS		
	ELS		
Microscope	AFM	Morphology	(52, 53)
	TEM		
	SEM		
Others	NMR	Thermal stability, etc.	(19, 54, 55)
	DSC		
	TGA		

CE, Capillary electrophoresis; SDS-PAGE, Sodium dodecyl sulfate polyacrylamide gel electrophoresis; MALDI-MS Matrix-Assisted Laser Desorption/Ionization Time of Flight Mass Spectrometry; ESI-MS, Electrospray Ionization Mass Spectrometry; FT-IR, Fourier Transform infrared spectroscopy; CD, Circular Dichroism; SEC, Size exclusion chromatography; SLS, Static Light Scattering; DLS, Dynamic Light Scattering; ELS, Electrophoretic light scattering; TEM, Transmission Electron Microscope; AFM, Atomic Force Microscope; NMR, Nuclear Magnetic Resonance; DSC, Differential scanning calorimetry; SEM, Scanning Electron Microscope; TGA, Thermogravimetry Analysis.

### 3.1 Electrophoresis

In an applied electric field, electrophoresis can be used to separate proteins by differences in charged molecules based on their conformation, molecular weight, isoelectric point and spatial structure (59). SDS-PAGE is a technique used for the separation of proteins according to their molecular weight and charge. SDS-PAGE electrophoresis along with both β-mercapto-ethanol and SDS can break non-covalent bonds between proteins and other substances, but not the covalent bonds. Therefore, SDS-PAGE can distinguish between covalent and non-covalent complexes of protein (60). Liu et al. characterized the covalent complexation between whey proteins and polyphenols by the SDS-PAGE technique, while the degree of conjugation of different polyphenols with whey proteins varied (40). Also, SDS-PAGE was used to evaluate the covalent complexation process of ovotransferrin and catechin induced by using free radical and treatment methods (61). Besides, Tao et al. showed that beta-lactoglobulin (β-LG) dimerization occurred after EGCG covalent modification by SDS-PAGE analysis (36).

Capillary electrophoresis (CE) is a chemical analytical technique that involves the separation and detection of sample components through the use of a capillary tube as a separation channel, in conjunction with an electrolytic bath to facilitate the process. The primary disadvantage of CE is that sample adsorption results in diminished separation efficiency and sensitivity. Polyphenol-protein complexes can also be distinguished from free proteins through the utilization of CE. In addition, covalent complexes *found* in polyphenol-rich beverages, foods and beverages are mostly characterized through CE coupled with chromatography (41).

### 3.2 MS technique

The MS has a powerful analytical capability to provide stoichiometric and structural information on the covalent complexes of polyphenols with proteins based on changes in the molecular mass-to-charge ratio. It has been shown that MALDI-MS and ESI-MS are valuable tools for the structural characterization of protein covalent complexes. MALDI-MS method is a soft ionization technique that provides quantitative information insights into the average molecular weight and distribution of biopolymers (62). The integration of ESI-MS with solution-based establishment separation techniques, such as HPLC methods, enhances its utility as a robust tool for characterizing the structural and compositional properties of biopolymers (62). MALDI-TOF-MS method is a new type of soft ionization biomass spectrometry. The MALDI-TOF-MS method operates on the principle of first mixing the sample with a matrix to create a crystalline film, followed by irradiation with a laser. The matrix absorbs the laser energy and subsequently transfers it to the sample molecules, resulting in ionization through the acquisition or loss of protons during the ionization process, ultimately leading to the ionization of the molecule. The charged molecules undergo acceleration within the flight tube due to the presence of an electric field, with the ions being identified based on the varying time taken to reach the detector relative to their mass-to-charge ratio (M/Z), which is directly correlated to the flight time (63).

Qie et al. analyzed chlorogenic acid and β-LG covalent complexes using MALDI-TOF-MS, wherein high temperature could promote the formation of polyphenol-protein covalent complexes (43). Additional, microLC-timsTOF-Pro-MS/MS in combination with bioinformatics was employed to identify C160 as (−)-epicatechin *o*-quinone and the main covalent binding site of β-LG after protein-polyphenol reaction was analyzed in solution formed from incubation of β-LG with (−)-epicatechin at 37 and 60°C (42). The above covalent process offers great potential for a comprehensive food analysis strategy. The electrospray ionization-MS (ESI-MS) can be used to further confirm covalent bonding and assess molecular weight changes of the conjugates. Based on ESI-MS results, the researchers observed α-lactalbumin (ALA)-catechin covalent modifications and at least one catechin molecule bound to ALA (44). Prigent et al. found that the molecular weight of α-ALA and lysozyme increased from 680 to 690 Da with the addition of chlorogenic acid, thereby suggesting that the proteins were covalently bound to quinones (64).

### 3.3 Spectroscopy

Endogenous fluorescence spectroscopy can be used to characterize tertiary structural changes in proteins. Because proteins possess chromogenic groups such as tryptophan, tyrosine, and phenylalanine, the proteins themselves have endogenous fluorescence characteristics. Information on the conformational changes of proteins is obtained by detecting the chromophores of proteins. Ishtikhar and colleagues observed (46, 65) that when the excitation wavelength of the fluorescence spectrum of protein was 295 nm, the endogenous fluorescence of protein mainly came from tryptophan, while tyrosine and phenylalanine were not excited, but when the excitation wavelength was 280 nm, the endogenous fluorescence of protein mainly came from tyrosine and tryptophan, while phenylalanine was not excited.

The CD is currently the main method for the determination of the secondary structure of proteins, which have multi-chiral and photoactive properties such as peptide bonds, disulfide bonds and aromatic amino acid residues in their structures. It was shown through CD and fluorescence spectroscopy that EGCG could induce changes in different tertiary and secondary structures of soy protein before or after heating. During heating, EGCG rapidly react with exposed aromatic amino acids of the protein prior to aggregation of the macromolecule. After heating, addition of EGCG to soymilk caused increased disarrayed spirals of soy protein increased more, thereby leading to protein conformation that has more disordered structures (45). In addition, the covalent binding of camphor seed kernel proteins and phenolic compounds led to an increase in β-folding (from 19.81 to 21.39%) and a decrease in random coiling (from 26.07 to 24.87%) (47). The above results indicate that different polyphenols interact with proteins and have different effects on the secondary structure of proteins. Some scientists have provided insight into nature of molecular structural interactions and impacts of polyphenol types using UV–Vis spectroscopy. On this score, Liu et al. discovered that the structure of zein was altered by the formation of covalent compounds using UV–Vis spectroscopy (19).

### 3.4 Other analytical technologies

In addition to the above techniques, many other analytical techniques are available for the characterization of covalent complexes. Static light scattering (SLS), dynamic light scattering (DLS) and electrophoretic light scattering (ELS) techniques were used to characterize the changes in the physicochemical properties of proteins after binding to polyphenols (51). In combination with analysis, information on charge, conformation, hydration radius and molecular weight of biopolymers can be provided by these techniques, wherein the biopolymers can facilitate further development of complexes as active substance delivery systems (50, 66). Atomic force microscopy (AFM) measurements provide insight into complex morphology, aggregate formation, and the effect of polyphenol affixation on proteins. Of note, information on the shape and size of covalent complexes can be provided by the AFM technique. Nonetheless, AFM imaging uses dehydrated samples, hence the outcomes cannot be compared to those of DLS (hydrodynamic diameters) obtained in dispersions. Besides, differential scanning calorimetry (DSC) can be used to assess the thermal stability of protein-polyphenol covalently bound systems (19, 54). Since each of the above analytical methods has its own strengths and weaknesses, several of them should be used in combination to obtain reliable and desirable experimental results.

## 4 Bioactive effects of polyphenol-protein covalent complexes

### 4.1 Effect of polyphenol-protein covalency on potential polyphenol bioaccessibility

The effect of polyphenol-protein covalency on the bioaccessibility of polyphenols is currently unclear. The interaction of proteins with polyphenols can adversely affect the nutritional value and digestibility of proteins, mainly because the interaction disrupts the structure of essential amino acids. Rawel et al. showed that phenolic compounds, such as chlorogenic acid and caffeic acid, reduced the lysine, cysteine and tryptophan content of soy protein when the former were covalently combined with the latter, which affected biological availability of the aforementioned amino acids (67). In addition to this, there are favorable effects of protein-polyphenol interactions. Several studies have shown that the covalent formation of polyphenol-protein has positive or even paltry impacts on the bioaccessibility of phenolics. It was found that the addition of green coffee powder to bread recipes significantly increased the phenolic content, with the addition of 5% increasing the phenolic content by 4.17 times (68). However, it is believed that the phenols eventually separated from the polyphenol-protein complexes and then did not affect the total absorption of the phenols (69). Also, there was a significant influence on the functional properties and digestibility of proteins due to protein-phenolic interactions. It was found that chicken polyphenols were covalently bound to myofibrillar protein via ultrasound assistance, which increased the bioaccessibility of the protein (34). Research has indicated that when coffee and cocoa polyphenols were combined with β-LG, the digestion of β-LG by pepsin was delayed by a factor of 2 at pH 1.2. Following *in vitro* gastrointestinal digestion, the polyphenols could produce a phenolic layer that minimized protein digestion in the digestive system. This layer provided protection for digestive enzymes by preventing them from targeting the cleavage sites of the proteins, thereby boosting digestive stability (70).

Indeed, several protein-phenolic covalently linked complexes have been developed to improve the stability of food-grade emulsions or granules, bioavailability and bioaccessibility of interacting phenolic compounds, which emphasizes the effectiveness of the associated systems as innovative carriers of bioactive ingredients (71). In general, protein-polyphenol interactions affect protein and phenol bioaccessibility both positively and negatively, therefore, for a thorough understanding of this complex relationship, more studies are needed. Moreover, there are no *in vivo* experiments to study the bioaccessibility of protein-polyphenol complexes.

### 4.2 Effect on digestibility

Food are taken in through the oral cavity and undergo the processes of digestion and absorption within the oral cavity, stomach, and small intestine, facilitated by various enzymes. Therefore, it is nutritionally standpoint to understand the impact of polyphenol protein covalency on digestibility of protein. Jiang et al. (72) found that anthocyanins were covalently coupled to soy protein, and the hydrolysis of the coupling product in the 5 h simulated small intestine was close to 60%, whereas the hydrolysis of soy protein was only about 45%. This may be due to the fact that the alteration of the conformational structure of soy protein induced by the presence of anthocyanins, rendering it more vulnerable to hydrolysis by digestive enzymes (e.g., pepsin and pancreatic enzymes) (73). Waqar et al. (74) prepared β-LG covalent bound to 4-methylcatechol, observing an increase in digestibility after 2 h of pepsin digestion (3.7%) and after 4 h of pancreatic digestion (34%) in comparison to β-LG (2.9, 32%). These findings suggest that the presence of covalent bonds does not significantly impact protein digestibility.

In addition, Zhou et al. (75) investigated the effect of *in vitro* digestion of EGCG-SPI covalent and non-covalent on polyphenols. After simulated gastric and small intestinal digestion, the residual rate of EGCG in covalent and non-covalent was 95.30 and 74.3%, respectively, while the residual rate of EGCG in the control group was 64.01%, which indicated that the protein exhibits a protective influence on polyphenols, with the covalent attachment to EGCG demonstrating a heightened protective efficacy. While in this study, the protein digestibility of covalent and non-covalent was 54.00 and 59.14%, respectively, which was significantly lower than that of SPI (76.17%). Variations in research findings may be attributed to variances in the specific proteins and polyphenols utilized in the studies. It is also important to consider polyphenol-protein covalent interactions through simulated *in vitro* digestion studies in the colon, and to investigate the impact of these interactions on the colonic microbiota.

### 4.3 Effect on antioxidant activity

Usually, the combination of polyphenols with proteins protects and even enhances the antioxidant activity of polyphenols. Wang et al. utilized laccase as a catalyst for covalently binding soy protein with gallic acid. The resulting covalent compound exhibited a remarkably enhanced antioxidant capacity, which was found to be directly proportional to the concentration of gallic acid. Notably, the respective 2, 2-diphenyl-1-picryl-hydrazyl (DPPH) free radical scavenging rate and 2, 2′-azino-bis(3-ethyl-benzo-thiazoline-6-sulfonic acid) (ABTS) free radical scavenging capacity of the compound were nearly 5-fold and 1.5-fold higher than those of soy protein alone, while the reducing power was more than 3-fold higher (76). The covalent complex of catechin and OVA exhibited enhanced scavenging activity against DPPH and ABTS radicals and had a higher reduction capacity for ferric reducing antioxidant power (FRAP) (77). Covalent complexes of polyphenols that have been bound to different proteins displayed varying antioxidant activity, with OVA-EGCG showing the highest antioxidant activity (78). Also, available literature has suggested that coupling of β-LG and EGCG provided substantial protection against oxidation of low-density lipoprotein (LDL), which may beneficially suppress atherosclerosis (36). As a result of the covalent connection between catechin and lactalbumin, their scavenging activity was higher than that of proteins or polyphenols alone, thereby suggesting that when the two components were combined, a synergistic effect occurred (44). Moreover, the anticancer activity of whey protein combined with quercetin or onion extract was substantially enhanced against cell line of human lung cancer (H1299) (79). It has been discovered that interaction between proteins and polyphenols resulted in the introduction of active OH group from polyphenols into proteins, thereby conferring upon the proteins a significantly augmented antioxidant capacity (40, 80, 81). So that it can endow proteins with significantly increased antioxidant activity. This makes the polyphenol–protein complexes used as antioxidant emulsifiers, antioxidant films, etc. In addition, there are potential applications of the above-mentioned covalent complexes in the pharmaceutical and cosmetic fields.

### 4.4 Effect on antibacterial activity

Polyphenols have strong antibacterial effect, however, their powerful antibacterial mechanisms involve protein binding and modification, which are essential for the survival of bacteria. It was found that serum from athletes supplemented with blueberry-green tea-polyphenol soy protein complex significantly delayed the exercise-induced increase in viral replication (*p* < 0.05) (82). Previous studies have shown that polyphenols of black tea could interrupt the pathogenic cell membranes via binding to proteins of the membranes and form covalent complexes that may act in a bactericidal or bacteriostatic manner (83). Based on existing work, phenolics such as carvacrol and resveratrol-trans dihydro-dimer have been found to affect Gram-negative and-positive bacteria, wherein the authors observed disruption of membrane potential when resveratrol-trans dihydro-dimer was incubated with *Bacillus cereus* and *Escherichia coli*, amid the above process being regulated by cell division (84). This disruption occurs when phenolic OH groups interacted with proteins, thus leading to changes in protein conformation that culminate in membrane potential alterations (84). Besides, polyphenols of apple, specifically proanthocyanidin interacted with *staphylococcal enterotoxin A* protein (responsible for most staphylococcal-induced food poisoning, a toxin of *Staphylococcus aureus*), which has been reported to prevent production of the toxin (85). In covalent interactions, phenolics are converted to quinones, which then react with protein nucleophilic groups (i.e., NH_2_ and -SH). The structure of the polyphenols is altered and their structure–function relationship affects their antimicrobial activity.

### 4.5 Effect on hypoglycemic activity

Polyphenols found in foods of plant origin have shown therapeutic activity against chronic diseases such as type-2 diabetes. In a clinical trial, serum collection and metabolomic analysis of athletes who consumed blueberry and green tea polyphenols with soy protein isolate (SPI) complex for 17 days showed elevated 3-hydroxy-butyrate and acetoacetate with SPI complex consumption, thus indicating enhanced ketogenesis and fatty acid oxidation during 3 days of exercise recovery (86). Roopchand et al. found that 300 and 500 mg/kg doses of grape pomace polyphenol-SPI complex significantly reduced blood glucose in obese and hyperglycemic C57BL/6 mice at 6 h after administration, with the 500 mg/kg dose approaching the effect of metformin (87). Also, 300 and 600 mg/kg doses of defatted soybean meal complexed with grape polyphenols significantly reduced blood glucose levels in hyperglycemic C57BL/6 J mice (88). Ribnicky et al. demonstrated that complexation of soy protein with polyphenols of *Artemisia dracunculus* L. increased the acute hypoglycemic activity and improved the bioavailability of polyphenols (89). The complex improves the therapeutic effect of polyphenols in type-2 diabetes, possibly because the protein-bound polyphenols are protected during gastrointestinal transit allowing more of the compounds to be maintained in the small intestine and potentially active, or allowing more polyphenols to be transported to the colon for microbial metabolism into other active metabolites. Thus, polyphenol-rich proteins may provide a new food system for creating nutrient-rich, low-sugar and high-protein food compositions that can be used in the dietary management of diabetes or metabolic syndrome.

### 4.6 Effect on anti-inflammatory activity

A consistent correlation has been shown between diet and inflammation (90). Dietary polyphenols have been extensively used in the inhibition of inflammatory responses and, in addition, some proteins and peptides have exhibited anti-inflammatory effects.

Blueberry polyphenol extract-protein aggregates significantly reduced RAW 264.7 cellular reactive oxygen species (ROS) production and down-regulated gene expression of inflammatory markers (COX-2 and IL-1β), in addition to inhibiting nitric oxide (NO) production and gene expression of inducible nitric oxide synthase (iNOS) (91). Zein-based resveratrol nanoparticles provided high and prolonged plasma levels of polyphenols and reduced serum tumor necrosis factor-α (TNF-α) levels in an lipopolysaccharide (LPS)-induced endotoxin shock model mouse for at least 48 h (92). Silk sericin hydrolysates and oxidized flavonoids form covalent that were significant in inhibiting NO and 15-lipoxygenase (15-LOX) production (93). Our hypothesis is that proteins have the capability to encapsulate polyphenols, thereby protecting and enhancing their stability (94). In summary, anti-inflammatory activity can be increased by the interaction of specific proteins with polyphenols, especially through covalent bonding.

### 4.7 Effect on modulation of gut microbiota

An increase in oxidative stress can result in abnormalities in flora of intestines and changes in body pathology. Natural endogenous hormones, active constituents and novel functional nanocarriers controlled the oxidative stress response, wherein proteins and polyphenols are vital regulators. These ingredients can play a significant role to maintain microecology of intestines and regulate the intestinal flora species composition by promoting antioxidant enzyme activity and effectively reducing free radicals in the body (95, 96). Increased polyphenols bioavailability, promotion of protein digestion and absorption and enhanced total antioxidant capacity are the potential benefits of proteins-polyphenols interaction (97, 98). Current studies have shown that small-molecule protein and polyphenols synergistically suppressed aging and regulated intestinal bacteria (99). The addition of mung bean protein-polyphenol complexes aided *Roseococcus*, *Bifidobacterium* and *Bacteroidetes* proliferation in intestines of senescent mice and inhibited *Bifidobacterium* and *Roseburia* growth, amid the complexes being superior to mung bean protein in the proliferation of *Bifidobacterium* (100). Protein protection may enhance the bioavailability and stability of polyphenols. Polyphenols can enter the colon through the upper gastrointestinal tract and are more readily absorbed and transported by the monomolecular layer (101). In future, protein-polyphenol complex foods can be developed to promote the microecological balance of intestinal flora.

### 4.8 Effect on modulation of allergic reactions

Food allergies do not only have a negative impact on quality of life, but can also have life-threatening consequences. It is estimated that 90% of food allergies are caused by milk, eggs, peanuts, nuts, shellfish, fish, wheat or soy (102). Several studies have shown that the interaction of polyphenols with proteins affects the sensitization of proteins. The results of established studies have confirmed that complexation of anthocyanin- and proanthocyanidin-rich polyphenol extracts with peanut matrix demonstrated substantially decreased binding capacity of immunoglobulin (IgE) and suppressed release of histamine and β-hexosaminidase from mast cells (103, 104). Also, existing studies have shown that the complexation of these polyphenols with proteins is covalent in nature (105). In contrast to the significantly lower IgE binding capacity of these EGCG-OVA conjugates compared to natural OVA, while changes in protein structure was induced by noncovalent complexes of EGCG-OVA but did not reduce interactions of IgE allergen owing to low affinity (106). Also, Wu et al. successfully reduced the sensitization of β-LG by covalently binding it to EGCG and chlorogenic acid (107). Various studies have found that the allergenicity of soy protein was reduced by the covalent action of polyphenols on soy 7S protein (108, 109). *In vivo* studies also showed that complexation of peanut protein-polyphenol decreased plasma IgE in C3H/HeJ mice that have been sensitized by peanut, in addition to a significant downregulation of CD63 expression (a surface marker protein of basophil), thus implying a reduction in allergic reactions in the above-mentioned mice (110). These studies provide a new approach to reducing protein allergenicity in food industry production. Binding of proteins to polyphenols leads to structural changes in the proteins that may eliminate conformational IgE-binding epitopes, thereby affecting the sensitization of the proteins. To further confirm these results, the “complex structural sensitization” relationships behind certain food allergens need to be elucidated and the mechanisms should be studied in greater depth.

**Figure 3:**
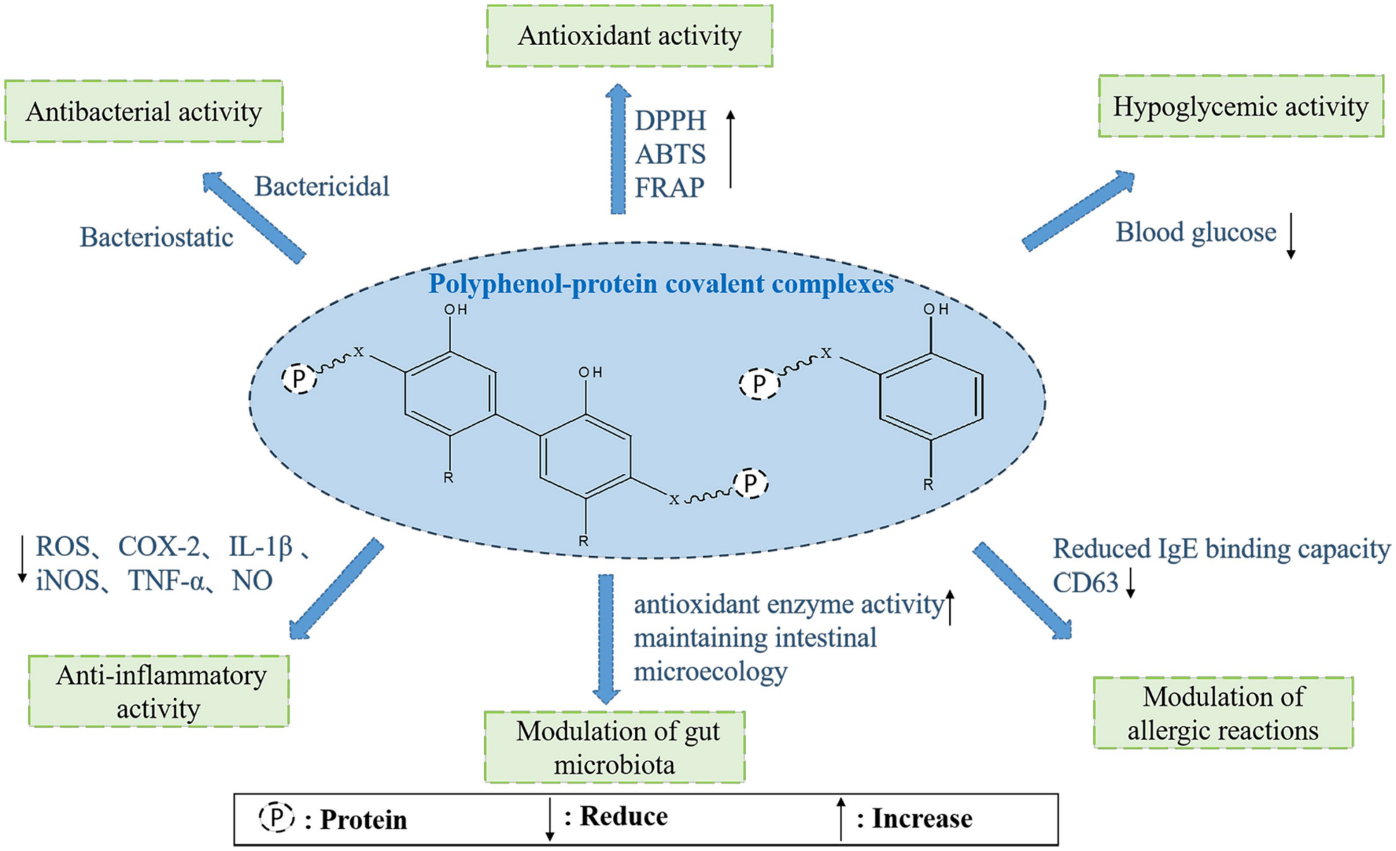
Biological activity of polyphenol-protein covalent complexes.

A schematic representation of the possible biological activities of polyphenol-protein covalent complexes is displayed in Figure 3. Current studies on the activity of protein-polyphenol covalent are mainly *in vitro* experiments, and only allergenicity has been studied more *in vivo* in experimental animals. Therefore, future *in vivo* preclinical and even clinical studies are needed to investigate the activity and mechanistic action of the complexes.

## 5 Polyphenol-protein covalent complexes in food applications

### 5.1 Emulsifiers agents

Protein-polyphenol covalent complexes have a wide range of applications in emulsion delivery systems because of their good antioxidant and emulsifying properties. The complex showed better advantages as an emulsifier compared to the single control protein. For instance, the covalent bond between catechin-α-ALA exhibited superior stability, with a particle size increase of less than 10% after 30 days of storage at 25°C (44). After being stored at 37°C for 30 days, the β-carotene content in egg white proteins decreased by approximately 15%. However, in the catechin-egg white protein covalent, the degradation of β-carotene was approximately 60% slower (111). Furthermore, it has been reported that covalently bound emulsions of catechins with β-LG and lactoferrin can effectively encapsulate β-carotene (112, 113). Abd et al. prepared β-LG-caffeic acid covalent complexes by using carbodiimide crosslinkers as functional emulsifiers with antioxidant properties. Meanwhile, the results showed that the covalent complexes did not only demonstrate better water solubility, but also significantly improved the antioxidant properties and stability of the emulsion as well as reduced the oxidation of lipids in the emulsion. As compared to β-LG (% scavenging index = 34.2), β-Lg- caffeic at pH 6 and pH 8.5 was a more potent scavenger of DPPH radicals (% scavenging index = 88.9 and 91.6, respectively) (35). The preparation of stable emulsions from covalent complexes exhibited great potential advantages, mainly as follows (56, 114, 115). (1) Using covalent complexes, each bioactive substance could effectively play its respective functionalities, such as proteins acting as surfactants and polyphenols as antioxidants, or the two working together. (2) Covalent complexes can be used to reduce the effects of environmental factors such as temperature, pH, ionic strength and enzyme catalysis on emulsions and improve emulsion stability. For example, the denaturation temperature of linseed protein-hydroxytyrosol covalent (154.30°C) was significantly higher than that of flaxseed protein (147.06°C), which may be related to the conformational change of the protein upon covalent modification with phenolic compounds (26). The improved emulsification activity is attributed to the increased surface hydrophobicity of the protein after modification, while the improved emulsion stability is attributed to stronger repulsive forces between the droplets encapsulated in the covalent complex (116). Another report showed that the foaming and emulsifying properties of lentil protein decreased after interaction with red onion peel phenolic extracts/gallic acid, but the bioavailability or antioxidant capacity of the phenolic compounds increased significantly (117). It is essential to consider the type, while content and ratio of phenolics and proteins should be fully considered when designing functional foods since they interact with one another and modify their functional properties and biological activity. As well, covalent complexes are limited in their application due to their complex, time-consuming and expensive preparation process.

### 5.2 Foaming agents

Available literature has stated that the interfacial properties of proteins and their capability to form and stabilize foams were affected by generation of polyphenol-protein complexes (118, 119). Studies on various whey protein (WP, like lactoferrin, α-LA and β-LG) that have been covalently associated with EGCG has shown that the resulting conjugates display superior interfacial properties in comparison to noncovalent complexes of WP-EGCG (116). For example, the interfacial protein adsorption fractions of α-LA, noncovalent α-LA-EGCG complex, and covalent α-LA-EGCG complex were 69.9, 66.1, and 77.1%, respectively; and the interfacial protein adsorption fractions of β-LG, noncovalent β-LG-EGCG complex, and covalent β-LG-EGCG complex were 81.4, 75.9, and 88.9%, respectively. The disparity in the strength of binding interactions between proteins and polyphenols in covalent and noncovalent complexes likely accounts for the ease of separation observed. Specifically, the strong interactions present in covalent complexes result in a higher degree of hydrophilicity on the protein surface compared to noncovalent interactions. Furthermore, the formation of covalent complexes can result in the development of an interfacial layer on the oil droplet’s surface, ultimately leading to enhanced stability (116).

A significant increase in foaming properties has been reported for whey proteins that have been covalently bound to chlorogenic acid (120, 121), tannic acid and sodium caseinate (122) as well as egg proteins that were covalently bound to green tea polyphenols (123). It was reported that the conjugation of gallic acid and EGCG with whey protein enhanced their foaming properties, which can be attributed to the increased molecular flexibility caused by the conjugation (124). The foaming ability is closely related to the rapid diffusion of proteins toward the air-water interface. Baba et al. used ultrasound treatment to link whey protein with quercetin by covalent bonding, which resulted in significant increased foaming capacity over whey protein (125). Lin et al. (108) showed that covalent modification of soybean 7S protein with chlorogenic acid and EGCG increased the foaming properties from about 15% to about 30%, with foam stability above 80%. The covalent foaming ability of ovalbumin cross-linked using laccase-catalyzed ferulic acid was higher (99.7%) than that of natural ovalbumin (94.3%) (126). The foaming performance of the system was influenced by factors such as protein adsorption at the air-water interface, the amount of adsorbed protein, and conformational changes at the interface (127). Protein-polyphenol complexes therefore exhibit different foaming properties depending on polyphenol and protein nature and structure as well as coupling method, and thus further research is needed to determine the best binding methods between different proteins and polyphenols in order to produce new compositions with improved functional properties.

### 5.3 Nanomaterials

**Figure 4:**
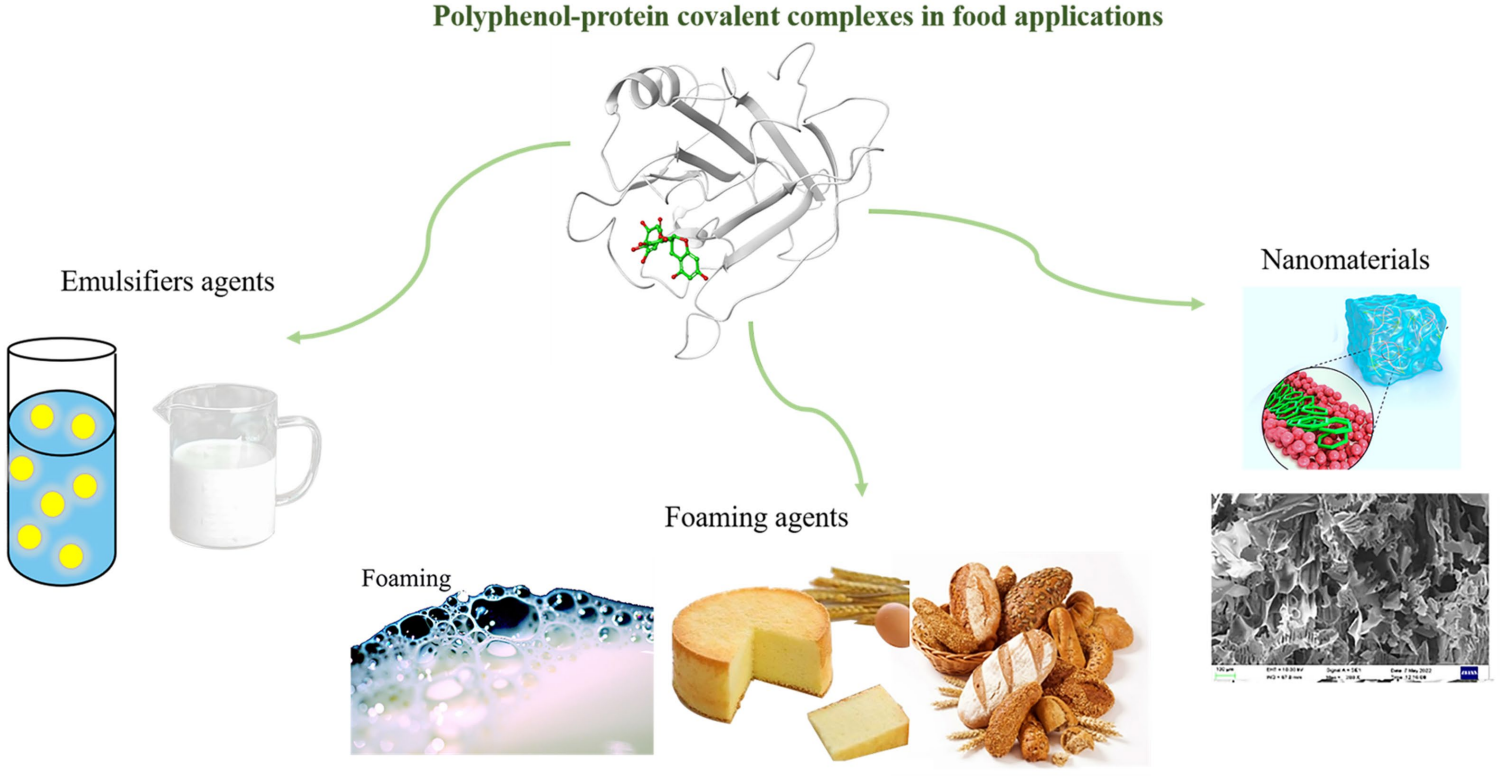
Polyphenol-protein covalent complexes in food applications (128, 135).

In addition, protein-polyphenol covalent complexes can also be used as nanomaterials. Because proteins are of amphipathic nature, complexes of protein-polyphenol have binding affinity for both hydrophilic and hydrophobic substances (128–130). Therefore, the use of phenolic-protein adducts as new carriers for unstable bioactive substances (namely phenolic compounds, fatty acids, hormones, other lipophilic and drugs) has recently become a hot topic (131, 132). Ge et al. prepared corn protein-soybean seed coat polyphenol covalently cross-linked nanoparticles, which improved the oxidative stability of soybean seed coat polyphenols and reduced the release of free fatty acids during simulated *in vitro* gastrointestinal digestion (133). Lau et al. discovered that protein-polyphenol complexes have great potential for oral administration of a variety of active food-derived ingredients through several experiments, including intestinal and this was accomplished by using protein-polyphenol complexes as potential materials for multilayer membrane microcapsules (134). Polyphenols and proteins can be covalently bound without chemical cross-linking agents, while both studies on complex properties demonstrated better functional advantages, followed by superior properties in comparison with single protein emulsions in delivery systems. Hence, theoretically polyphenol-protein covalent have great potential as functional complex carriers. A schematic representation of the possible applications of polyphenol-protein covalent complexes in foods is presented in Figure 4.

## 6 Summary and future perspective

Current research works clarifying the mechanism of action of polyphenol-protein covalency was highlighted in this review, while the impact of various food processing methods, characterization techniques, health-promoting effects, properties of covalents, and their applications in the food field were discussed. Polyphenols exert an influence on proteins with regard to their solubility, thermal stability and other related characteristics. These functional properties are essential to determine the physical properties of food ingredients during preparation, processing or storage. Thus, covalent binding of proteins and polyphenols is a potential approach to improve the stability of proteins subjected to thermal processing.

Scientists have achieved much progress in strengthening and clarifying the formation of polyphenol-protein covalent complexes through prevalent utilization of molecular docking for predicting phenolic compounds and protein binding sites (136). However, limited research has been conducted on comprehensively determining the binding sites and identifying the specific amino acid residues that directly contribute to the formation of polyphenol-protein covalent complexes. Furthermore, the impact of varying conditions such as temperature, pressure, and other factors during food processing on the alteration of these binding sites remains largely unexplored. The investigation of the formation of polyphenol-protein covalent complexes and the identification of specific binding reaction characteristics can be further enhanced through a practical approach. This refinement aims to assess validity of the universal law governing polyphenol-protein covalent complexes and facilitate later advancement of nutraceuticals and functional foods. Furthermore, there exists a limited body of research pertaining to the intrinsic characteristics of the covalent bond, which encompasses physicochemical attributes, biological functionality and various other properties.

Moreover, the utilization of model polyphenolic compounds-proteins has been prevalent in contemporary research on covalent interactions between polyphenols and proteins. Based on these aforementioned considerations, it is imperative to conduct further investigations pertaining to covalent interactions within real systems that encompass phenolic compounds, such as those found in tea, coffee, cocoa, etc., as well as proteins present in milk, soy, rice, etc. The findings of such studies, namely current study and previous works, may act as a reference for nutraceuticals and functional foods development, which may be utilized as efficacious candidates for monitoring the characteristic changes in polyphenol-protein covalent complexes.

*In vitro* findings have recently disclosed the nutritional value of polyphenol-protein covalent complexes; however, further investigation is needed to ascertain whether these nutritional values were detected at doses that are close to *in vivo* dietary intake levels. Further *in vivo* studies, as well as potential toxicity investigations should be strengthened for understanding the application direction of polyphenol-protein covalent complexes.

Altogether, the present perspectives provide new insights into formation and changes in polyphenol-protein covalent complexes and how polyphenol-protein covalent complexes improve health status, which offers new prospects for research trends in future and opportunities to boost development of functional food products toward precision nutrition.

## Author contributions

KZ: Conceptualization, Writing – original draft. JH: Conceptualization, Investigation, Project administration, Writing – review & editing. DW: Formal analysis, Methodology, Writing – review & editing. XW: Resources, Supervision, Visualization, Writing – review & editing. YW: Funding acquisition, Writing – review & editing.
